# Validity of a reactive agility test for aerobic and anaerobic performance assessment in female handball players

**DOI:** 10.3389/fspor.2026.1870795

**Published:** 2026-07-21

**Authors:** Nadir Szabo, Khatija Bahdur, Beatriz Jordão, Thorben Hülsdünker

**Affiliations:** 1 Department of Sport, LUNEX, Differdange, Luxembourg; 2 Luxembourg Health & Sport Sciences Research Institute (LHSSRI), Differdange, Luxembourg

**Keywords:** change of direction, cognition, diagnostics, ecological validity, endurance, maximum oxygen uptake, motor-cognitive, team ball sport

## Abstract

**Introduction:**

Aerobic and anaerobic capacity are considered key performance indicators in team ball sports. However, diagnostics focus on treadmill testing or linear sprints, not considering multidirectional change of direction and reactive agility. This study evaluated the validity of a novel multidirectional reactive agility test (RA test) to determine aerobic and anaerobic capacity in high-level female handball athletes.

**Methods:**

Fourteen first-team female handball players performed four all-out 150 m multidirectional RA runs on the SKILLCOURT. Maximum oxygen uptake (VO_2max_) was determined using a mobile gas analyzer and compared to an incremental ramp test on the treadmill. For anaerobic performance, peak power output (PPOmax), average power output (PPOav), fatigue index (FI) and anaerobic capacity (AC) were calculated based on running time in the RA test and correlated to the repeated anaerobic sprint test (RAST).

**Results:**

Bland-Altman analyses revealed a mean difference in VO_2max_ between the RA test and the treadmill test of 0.07 mL × kg^−1^ × min^−1^ (0.1%) with upper and lower limits of agreement (LoA) at 4.03 (8%) and −3.88 (8%) mL × kg^−1^ × min^−1^, respectively. Measured VO_2max_ values from the RA test and treadmill were strongly related (r = 0.91) and not significantly different (*p* = 0.90, d = 0.04). No significant correlations were found for any of the anaerobic variables (*p* ≥ 0.40).

**Discussion:**

The results support the suitability of a RA test protocol for directly measuring and estimating aerobic capacity (VO_2max_). This was not confirmed for anaerobic parameters. The RA test may provide a promising sport-specific alternative to existing lab and field protocols for determining VO_2max_ in team ball sports.

## Introduction

Despite the growth in participation rates in women's sports, the implementation of evidence-based practice remains limited and women remain underrepresented, with only 7% of research focused on female-only cohorts (1). Considering that also in mixed-gender athlete samples, females only account for 35%–39% of participants, sex-specific physiological demands and performance analysis for female athletes remain insufficiently understood (2). Also in handball, the existing literature has predominantly focused on male athletes or mixed cohorts, despite established sex-based differences in injury profiles (3), physical test performance, and physiological responses to match play (4). In fact, female players experience a higher relative workload during matches than male players (4). Creating recommendations and guidelines from male-only or mixed male–female cohorts failed to factor in not only biological, but also psychosocial, and socioeconomic factors (5), which may result in players being less exposed to high levels of training, scientific development, and being younger when playing senior handball. Given these differences, it is imperative that performance tests are validated in female players (6), enabling the use of sports assessments tailored to female physiology and the women's game.

Handball is an intermittent sport that induces neuromuscular fatigue owing to the frequency of accelerations, decelerations, change of direction (CoD) actions, sprints, jumps, and throwing actions (7). The intermittent nature of handball is reflected in physiological demands, with players operating at approximately 80% of VO_2max_ (8, 9). Recent rule changes have further intensified the game, leading to more high-intensity actions and shorter recovery periods (7). International female players cover a notable proportion of their total distance at high intensity, with 17.92% of the total distance as high-intensity running and 3.69% as sprinting (5) and perform an average of 2.90 high-intensity actions per minute (10).

These characteristics set particularly high demands on the aerobic and anaerobic energy metabolism, which are important performance indicators in team ball sports including handball (11). To determine VO_2max_ an incremental treadmill protocol is still considered the gold standard, although it has been criticized for its lack of ecological validity (12, 13). As an alternative, field tests such as the Cooper test (14), Yo-Yo Intermittent Recovery Test (YYIRT) (15) and 30-15 Intermittent Fitness Test (30-15 IFT) (16) have been developed to incorporate a game like environment, intermittent recovery and bidirectional CoD, making them more ecologically valid and popular tools in team ball sports. However, these tests do not include multidirectional CoD as well as reactive agility, two key components integral to handball. In fact, it has been argued that tests integrating motor and cognitive actions within a sport-specific context offer greater ecological validity and are more effective indicators of sport-specific fitness than generic, pre-planned assessments (17). Recent attempts to bridge this ecological gap have leveraged technology to create sport-specific assessments. Technologies such as SKILLCOURT (18) and SpeedCourt (12) have produced validated and reliable tests for different fitness components that are important in ball sports. Born et al. (12) introduced an incremental test on the SpeedCourt platform that was developed based on the YYIRT. It incorporated reactive agility as changes of direction in response to a visual stimulus and demonstrated reasonable limits of agreement with the gold standard treadmill test for VO_2max_ measurement. These findings suggest that a RA test protocol is feasible to elicit a VO_2max_ response. However, the reported correlation between the treadmill test and RA test protocol was comparatively low (r = 0.59) questioning the reliability of the measured VO_2max_ values. Furthermore, unlike other field-based tests, no behavioral performance parameter was available that allows estimation of VO_2max_. This is particularly important for coaches and athletes who do not have access to a gas analysis system but want to estimate the athlete's aerobic performance.

To address these limitations and incorporate sport-specific multidirectional reactive agility demands into VO_2max_ testing, the reactive agility test (RA test) using the SKILLCOURT technology has been developed. When compared to the test introduced by Born et al. (12), the RA test on the SKILLCOURT used four all-out multidirectional reactive agility runs of 150 m with 30 s breaks in between. This results in a test duration of about 10 min which is within the recommended range for VO_2max_ assessment between 8 and 12 min (19). Recent results confirmed the validity of directly measuring a true VO_2max_ response as well as a useful estimation of VO_2max_ based on overall running time comparable to established tests such as YYIRT (20). However, while this study was performed with athletes from different disciplines, it remains unclear if these results can be validated in a high-level and homogenous female-only sample. Moreover, it needs to be established if in addition to aerobic performance indicators, the test also allows determining anaerobic capacity due to its high intensity, all-out profile. While the repeated anaerobic sprint test (RAST) is often used as a more sport-specific alternative to the Wingate test (21) its linear sprint characteristics lack the multidirectional change or direction and reactive agility components essential in handball. The RA test on the SKILLCOURT may provide a more sport-specific alternative also for anaerobic performance testing due to its short sprint and change of direction demands.

Therefore, this study evaluated the validity of an RA test for determining aerobic and anaerobic performance indicators in female team ball sports. The specific demands of women's handball, including multidirectional changes in direction, sprints lasting 7 m–19 m, brief recovery periods (average 55 ± 32 s between high-intensity actions) (22), and the prevalence of motor-cognitive actions, support the use of RA based testing in this sport. First team female handball players were tested in a 4 × 150 m RA test and an incremental treadmill test while oxygen uptake was continuously measured. Moreover, data from Cooper test, 30-15 test and RAST were used to identify the relation between performance in the RA test and field-based indices of aerobic and anaerobic performance. In line with a previous study in a mixed gender sample (20), it was hypothesized that the RA test provides a valid measure of VO_2max_ and allows VO_2max_ estimation based on overall running time that is at least at the level of established Cooper test and the 30-15 Intermittent Fitness Test (IFT). Similarly, due to the reactive agility and all-out nature of the test, significant relations to anaerobic performance metrics derived from the RAST were expected.

## Methods

### Sample size calculation

The sample size calculation was conducted based on the previous study initially validating the RA test on the SKILLCOURT in a mixed gender and multisport sample (20). In this study, a correlation coefficient of r = 0.74 was observed between estimated VO_2max_ from the RA test and the measured treadmill VO_2max_. As the present study recruited a more homogenous sample of only female high-level athletes, a lower correlation coefficient was expected. Therefore, the sample size calculation was based on a correlation coefficient of r = 0.65 in a one-tailed bivariate correlation with an alpha level of 0.05 and statistical power of *ß* = 0.8 which resulted in a required sample of 13 participants. With 17 athletes tested and 14 in the final analysis, this study was considered sufficiently powered.

### Participants and ethics

Seventeen participants were originally recruited for this study. Two participants were excluded for not meeting the criteria for VO_2max_ in the treadmill test, and one participant was excluded for failing to complete the RA test. The final sample consisted of 14 female participants (age, 22.5 ± 4.4 years; height, 1.70 ± 0.05 m; weight, 70.7 ± 12.5 kg; VO_2max_, 48.4 ± 4.6 mL × kg^−1^ × min^−1^).

The participants were all highly trained and recruited from a national handball club, corresponding to tier 3 according to the classification of McKay et al. (23). The participants had an average of 14 ± 7 years of training experience and 6 ± 4 years of professional experience. The sample consisted of a goalkeeper (*n* = 1), left back (*n* = 2), center back (*n* = 2), left wing (*n* = 3), right back (*n* = 2), right wing (*n* = 2), and pivot (*n* = 2). Informed consent was obtained. The study was performed in accordance with the Declaration of Helsinki and was approved by the local national research ethics committee (nr. 202207/01 v2.0).

### Experimental protocol

**Figure 1:**
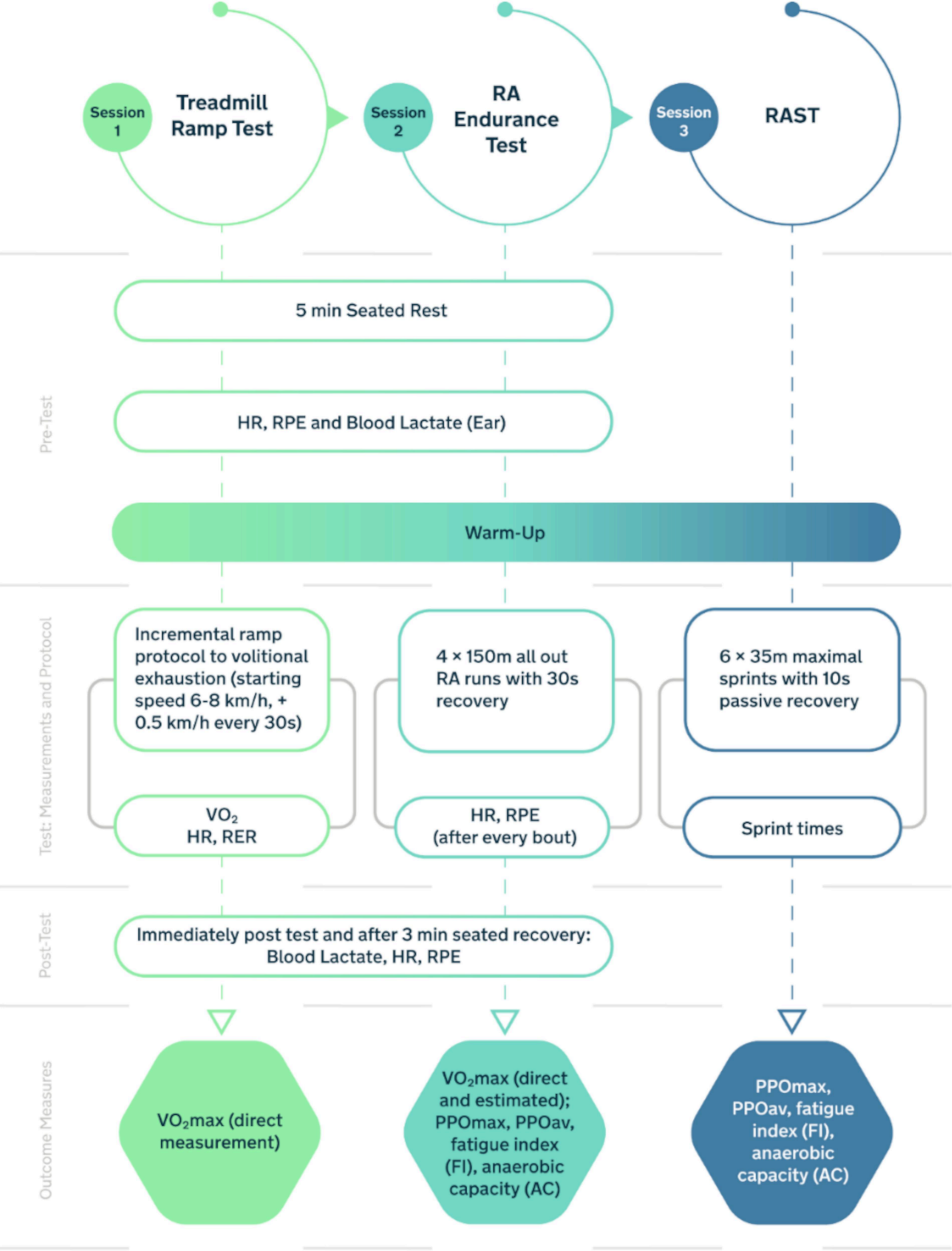
Overview of the experimental protocol.

Data collection for each participant occurred over three testing sessions, with the first two dedicated to measuring VO_2max_ (treadmill and RA test). On the third test day, anaerobic performance was determined using the RAST (24). Figure 1 illustrates the experimental protocol.

The treadmill and RA tests were performed at least 48 h apart. The participants were tested at the same time of day ±2 h to avoid circadian effects. The first day consisted of an incremental VO_2max_ test on a treadmill (h/p/cosmos®, Pulsar®, 133 Nussdorf, Germany) to volitional exhaustion. This was followed by familiarisation with the SKILLCOURT. On the second day, the participants performed the RA test on the SKILLCOURT (Skillcourt GmbH, Schweinfurt, Germany). Oxygen uptake, lactate, rate of perceived exertion (RPE; 6–20), and heart rate (HR) were measured in both tests. Oxygen uptake was continuously recorded breath-by-breath using a validated portable MetaMax 3B analyser (CORTEX Biophysik BmbH, Leipzig, Germany). Blood lactate samples were obtained from the earlobe before the test, immediately after and 3 min after finishing the test. Samples were analysed using a Biosen C-Line lactate analyser (EKF-diagnostic GmbH, Barleben, Germany). HR was measured throughout the test, using an H10 sensor, and a Polar Vantage V2 watch (Polar Elektro, Kempele, Finland). RPE was obtained verbally according to Borg's scale (25) prior to and after the treadmill test as well as after each run of the RA test*.* On the day of the RAST, the full sample of participants was tested consecutively within a period of 2.5 h. The anaerobic parameters that were derived were: 1) maximal power (PPOmax), 2) average power (PPOav), 3) fatigue index (FI), and 4) Anaerobic Capacity (AC). Verbal encouragement was provided in all testing sessions.

### Treadmill ramp test

Prior to testing, participants were instructed to perform a 5-min seated rest to normalise the resting values of physiological parameters. The goalkeeper started at 6 km × h^−1^ while all out-field players at 8 km × h^−1^ with a 1% incline (26), considering the different energy system demands that correspond to different handball playing positions (27). The treadmill test consisted of a 3-min warmup at the starting velocity, followed by an increase of 0.5 km × h^−1^ every 30 s. Once velocity reached 16 km × h^−1^, it remained constant, and inclination increased at a rate of 1% per minute (26). RPE, Lactate, and HR values were recorded following a 5-min seated rest, immediately after the exercise and after 3 min after exercise cessation. Oxygen uptake was continuously recorded using a portable gas analyser, and VO_2max_ was determined as the highest oxygen uptake (VO_2_) in an interval of 30 s. VO_2max_ was considered valid if the increase in oxygen uptake during the last minute did not exceed 150 mL (28). In the absence of a plateau, two of the four physiological indicators must be met: 1) HR ≥ 95% of maximum HR (220-age)) 2) blood lactate 8 mmol × L^−1^ 3) RER ≥ 1.1) 4) RPE ≥ 18 (20, 28). Two participants did not meet the criteria and were excluded. Following the treadmill test, participants were familiarized with the reactive agility exercise on the SKILLCOURT technology. Two 50 m reactive agility runs were performed at submaximal intensity.

### Reactive agility (RA) test

In the second session, the participants performed the RA test on the SKILLCOURT. The test consisted of four 150 m all-out RA runs with 30 s of rest in between. The participants were instructed to perform each trial at maximal effort without pacing. Prior to the test, the participants performed a 5-min seated rest. This was followed by a warm-up consisting of a run on the treadmill at self-selected velocity (5 min), dynamic stretching (5 min) and two 50 m RA runs at self-determined intensities of 60% and 80% with a three-minute resting period after each run. Subsequently, the RA test was initiated. During the test, the participants ran between eight target fields on a 4 m × 4 m court, corresponding to the fields indicated on a screen in front of them. The target field was indicated in yellow. Once the target field was reached, the next was presented. The distance was automatically calculated using a LiDAR (light detection and ranging). Consistent with the treadmill test, oxygen uptake was continuously recorded using the mobile gas analyser, and VO_2max_ was determined as the highest oxygen uptake in an interval of 30 s. Lactate samples were obtained following the 5-min seated rest, directly after the RA test*,* and following a 3-min seated cooldown period post-test. RPE was recorded post 5-min seated rest, prior to the test, at the end of each 150 m run, as well as at the end of the 3-min cooldown period post-test. HR was measured continuously throughout the test.

In addition to its direct measurement, VO_2max_ in the RA test was estimated using a regression equation (Equation 1) derived from the relationship between the total time in the RA test and treadmill VO_2max_ determined in a previous study (20).

(1) $$VO_{2\max}=0.1686\times \mathrm{RA}\;\mathrm{test}\;\mathrm{Total}\;\mathrm{Time}\;(s)+117.95$$

In addition to the estimation of aerobic parameters (VO_2max_), anaerobic performance indicators were calculated based on the running times in the four 150 m reactive agility runs. This included the peak power output (PPO) of all runs [Weight (kg) × Distance^2^ (m)/Time^3^ (s)], from which the highest (PPOmax), and average (PPOav) PPO values were derived. Further, the fatigue index (FI) [PPOmax − PPOmin/Total run time (s)], and anaerobic capacity (AC) [Σ(PPO)] were calculated in accordance with previous research (29).

### Repeated anaerobic sprint test (RAST)

On the third testing day, the RAST was performed with all participants. Prior to the test, each participant performed a 5-min warm-up consisting of submaximal runs and dynamic stretching. Each participant was thoroughly informed about the procedure. The test consisted of six 35 m sprints with 10 s of passive recovery in between (24). Participants were instructed to stop completely at the starting line prior to the start of the next sprint. The time of each sprint was measured using Witty wireless training timer (Micogate, Bolzano, Italy) timing gates. Consistent with the RA test, PPO, PPOmax, PPOav, FI, and AC were calculated as indicators of anaerobic capacity.

### Cooper test and 30-15 test

In addition to the RA test*,* VO_2max_ was estimated based on the results of the Cooper (14) and 30-15 intermittent fitness tests (30-15 IFT) (16). Both tests were not performed in this study but as part of athletes' regular performance assessments. The Cooper test was performed with the participants as part of preseason testing approximately 9 months prior to the study. The 30-15 IFT was performed approximately 4 months prior to the study. The VO_2max_ derived from the Cooper test was estimated in accordance with Cooper (14): VO_2max_ (mL × kg^−1^ × min^−1^) = (distance (m) − 504.9)/44.73. VO_2max_ was estimated from the 30-15 IFT according to Buchheit (16), considering the velocity achieved during the intermittent fitness test (VIFT), Age (A), Gender (G), and bodyweight (W): VO_2max_ (mL × kg^–1^ × min^–1^) = 28.3 − (2.15 × G) − (0.741 × A) − (0.0357 × W) + (0.0586 × A × VIFT) +(1.03 × VIFT).

### Statistical analysis

All statistical analyses were performed using JASP (version 0.19.3.0). Normal distribution was verified using Shapiro–Wilk tests. In case of violation, a non-parametric test was performed. Control analyses identified potential differences between resting physiological values (HR, LT, RPE) and starting times between the treadmill and RA test through a paired samples *t*-test.

Bland–Altman analysis assessed the limits of agreement in VO_2max_ measurements between the treadmill and RA test, while Pearson's correlation quantified the relationship between the two methods. The differences in VO_2max_ measurements obtained from the treadmill and the RA test were further examined using a paired samples *t*-test. These analyses were repeated to compare the measured treadmill VO_2max_ to 1) estimated VO_2max_ based on overall running time in the RA test and 2) VO_2max_ estimated based on the 30-15 IFT performance and 3) VO_2max_ estimated based on the Cooper test results. Correlation coefficients were compared using the Fisher's z test.

Pearson's correlation coefficient was implemented to study the relationship between the anaerobic variables: PPOmax, PPOav, FI and AC, derived from the RAST and with the RA test. Effect sizes were considered small (d = 0.2, r = 0.1), medium (d = 0.5, r = 0.3) or large (d = 0.8, r = 0.5). The significance threshold was set to *p* < 0.05.

## Results

The VO_2max_ values measured in the RA test and treadmill test as well as VO_2max_ estimated from the RA test, 30-15 IFT and Cooper test are summarized in Table 1. Control analyses did not reveal any significant differences in resting state physiological measures (HR, lactate, RPE) between the two test days (*p* ≥ 0.10), nor in the start times of the two tests (*p* = 0.29).

**Table 1:** VO_2max_ values elicited from the different tests.

Condition	Variables	Mean ± SD	*n*
Measured (VO_2max_)	Treadmill test	48.36 ± 4.62	14
	RA test	48.43 ± 4.88	14
Estimated (VO_2max_)	RA test estimated	50.19 ± 5.03	14
	Cooper test	39.32 ± 5.08	12
	30-15 IFT	48.74 ± 4.06	10

SD, standard deviation.

### Measured VO_2max_

**Figure 2:**
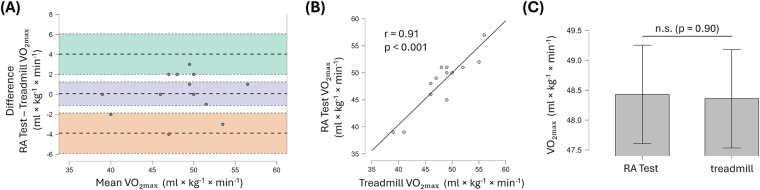
**(A)** Bland–Altman plots indicating mean difference and 95% limits of agreement between VO_2max_ elicited with the RA test and treadmill test, **(B)** Pearson's correlation scatterplot indicating a linear relationship between VO_2max_ elicited with the RA test and treadmill test, and **(C)** a paired samples *t*-test indicating the difference between the VO_2max_ values derived from the treadmill and RA test. Error bars indicate 95% confidence intervals.

The average test time of the treadmill test was 8.8 ± 1 min. The average test time of the RA test was 8.7 ± 0.5 min*.* Bland–Altman analyses revealed a mean difference between the RA test and the treadmill test of 0.07 mL × kg^−1^ × min^−1^ (0.1%), as well as upper and lower LoA of 4.03 (8%) and −3.88 (8%) mL × kg^−1^ × min^−1^, respectively. A strong relationship was identified between the VO_2max_ measured with the treadmill and RA test [r = 0.91 (CI: 0.74, 0.97), *p* < 0.001]. There was no significant difference in VO_2max_ measured in the RA test and the treadmill test (t = 0.13, *p* = 0.90, d = 0.04). The results are presented in Figure 2. The lactate levels at the end of the RA test and treadmill test were 8.59 ± 2.1 and 8.02 ± 2.6, respectively and not significantly different (*p* = 0.39).

### Estimated VO_2max_

**Figure 3:**
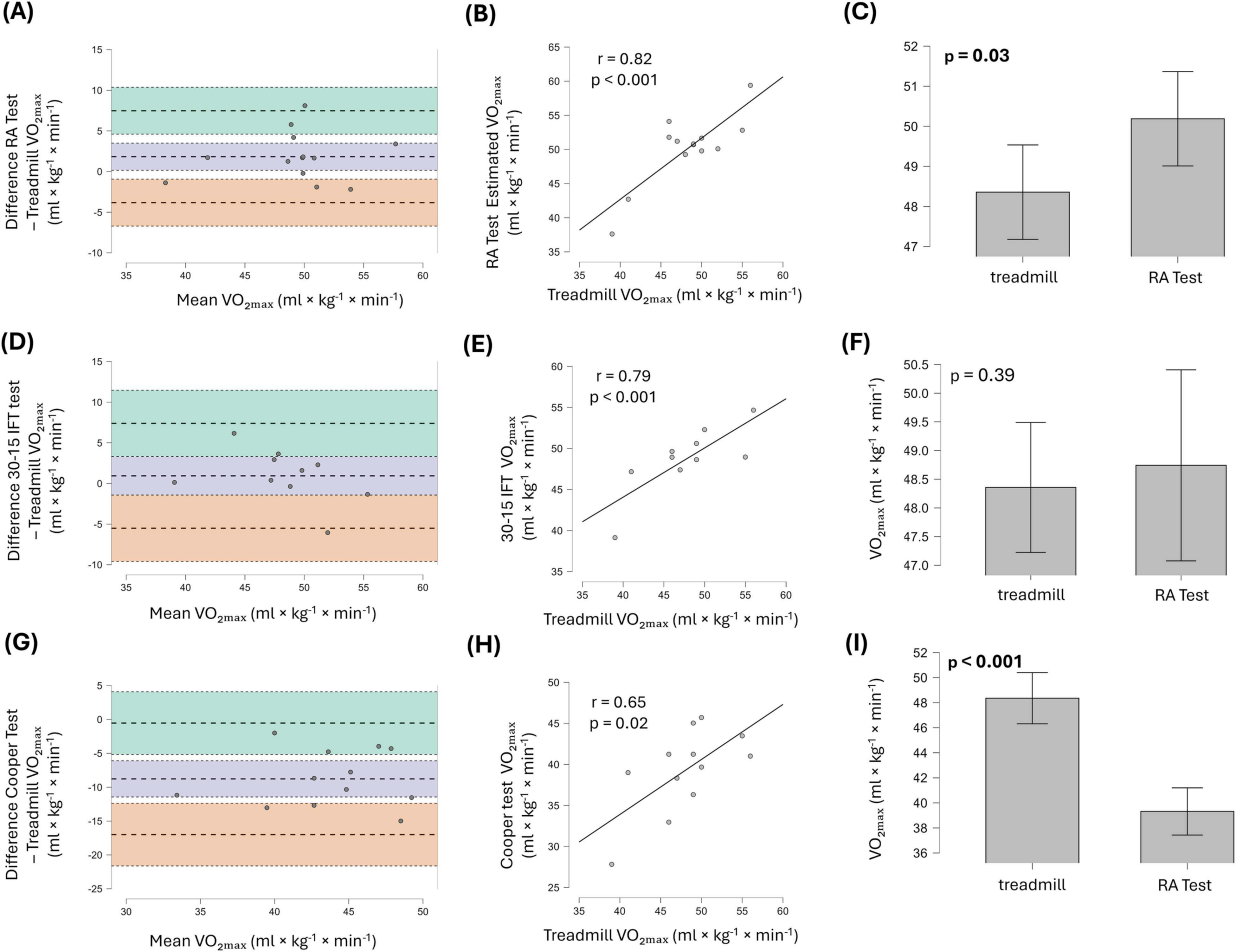
**(A)** Bland–Altman plot indicating the mean difference and limits of agreement between treadmill-based VO_2max_ measurement and estimated VO_2max_ from the RA test, **(B)** Pearson correlation scatterplot, indicating the relationship between both variables and a **(C)** *t*-test indicating the difference between VO_2max_ estimated with the RA test, and directly measured with the treadmill. **(D–F)** Similar analysis for VO_2max_ values estimated with the 30-15 IFT. **(G–I)** Similar analysis for VO_2max_ values estimated with the Cooper test.

Bland–Altman limits of agreement analysis comparing measured treadmill VO_2max_ with estimated RA test VO_2max_, revealed a mean difference of 1.83 mL × kg^−1^ × min^−1^ (4%), and upper and lower LoA of 7.49 mL × kg^−1^ × min^−1^ (15%), and −3.82 mL × kg^−1^ × min^−1^ (8%), respectively. Comparing treadmill VO_2max_ and VO_2max_ estimated by the Cooper test, resulted in a mean difference of −8.76 mL × kg^−1^ × min^−1^ (18%), and upper and lower LoA of −0.53 mL × kg^−1^ × min^−1^ (1%) and −17 mL × kg^−1^ × min^−1^ (35%), respectively. For the 30-15 IFT, the mean difference was 0.94 mL × kg^−1^ × min^−1^ (2%), with upper and lower LoA of 7.39 mL × kg^−1^ × min^−1^ (15%) and −5.51 mL × kg^−1^ × min^−1^ (11%), respectively. Pearson's correlation coefficient revealed significant correlations between treadmill VO_2max_ and all three VO_2max_ estimations [RA test: r = 0.82 (CI: 0.52, 0.94), *p* < 0.001; Cooper test: r = 0.65 (CI: 0.11, 0.89), *p* = 0.02; 30-15 test: r = 0.79 (CI: 0.32, 0.94), *p* < 0.001]. Fishers z-test revealed no significant difference between correlations (*p* ≥ 0.198). Paired samples *t*-tests revealed significant differences between treadmill VO_2max_ and VO_2max_ values predicted from the RA test (t = −2.38, *p* = 0.03, d = −0.63), and the Cooper test (t = 7.23, *p* < 0.001, d = 2.09). No significant difference was found for the 30-15 IFT (t = −0.91, *p* = 0.39, d = −0.29). These results are illustrated in Figure 3.

### Anaerobic parameters

**Figure 4:**
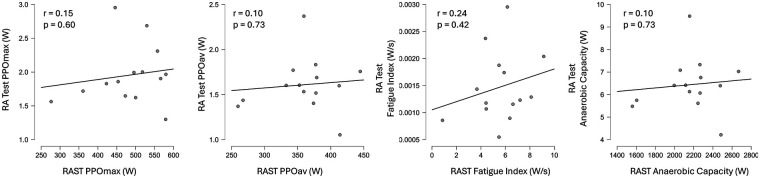
Relationship between anaerobic performance indices derived from the RA test and RAST illustrated (from left to right) for peak power output (PPOmax), average power output (PPOav), fatigue index (FI) and anaerobic capacity (AC).

Pearson correlation analyses for the anaerobic performance indicators are illustrated in Figure 4. There was no significant relation between the RAST and RA test in any of the anaerobic parameters, (PPOmax: r = 0.15, *p* = 0.60; PPOav: r = 0.10, *p* = 0.73, FI: r = 0.24, *p* = 0.42; AC: r = 0.10, *p* = 0.73). Descriptive statistics of the variables derived from the RAST are as follows: PPOmax was 510.07 ± 54.1 W, PPOav was 377.18 ± 32.4 W, FI was 6.13 ± 1.48 W/s, and AC was 2263.08 ± 194.45 W. For the RA test, the following values were observed: PPOmax was 2 ± 0.46 W, PPOav was 1.64 ± 0.3 W, FI was 0.0015 ± 0.31 W/s, and AC was 6.58 ± 1.23 W. Note that due to differences in running profile (linear sprint in the RAST vs. reactive agility in the RA test), there are large differences in the absolute values.

## Discussion

This study evaluated the concurrent validity of the RA test performed on the SKILLCOURT in elite female handball players to determine aerobic and anaerobic performance indices. The findings support the RA test's validity as an alternative method for measuring VO_2max_ in trained female handball athletes, with measurements in close agreement with the gold standard treadmill test. Estimated VO_2max_ values were of lower precision but at the same level or higher as the established Cooper test and 30-15 IFT. The RA test appears to be not suitable to determine anaerobic performance metrics.

With limits of agreement of about ±8% for the direct measurement of VO_2max_ in the RA test, this assessment provides a reasonable variability. This applies especially when considering a day-to-day variability in treadmill VO_2max_ testing of 5.6% (30) and technology-related variability of another 2% (31). Importantly, these LoA are in the same range as for the 30-15 IFT (LoA = 4.80 to −10.97) by Mohoric et al*.* (32) also investigating female handball players and substantially lower when compared to the widely used YYIRT (−26% to +16%) as reported by Castagna et al. (33). In addition, Mohoric et al. (32) reported a significant difference between VO_2max_ measured on the treadmill and in the 30-15 test (*p* = 0.007), as well as no relation between both values (r = 0.339, *p* = 0.155). In contrast, VO_2max_ measured in the RA test did not significantly differ from the treadmill-based VO_2max_ and showed a strong relation (r = 0.91). Also, for the reactive agility test on the SpeedCourt reported by Born et al. (12), the difference to VO_2max_ measured on the treadmill was substantially higher (3.1%) and LoA were larger (−10.8%–18%). Further, the correlation, although significant, was comparatively low (r = 0.59). These findings support the validity of directly measuring VO_2max_ in the RA test on the SKILLCOURT and supports its use as an alternative to lab-based treadmill tests or established field-based approaches.

For VO_2max_ estimation, the predicted VO_2max_ was associated with an estimated error of 4%, which is comparable to the 30-15 IFT (2%) and considerably lower than the Cooper test (18%). Nonetheless, LoA were substantially wider (−8% to –15%) when compared to the direct measurement of VO_2max_ in the RA test however narrower when compared to VO_2max_ estimation in a previous study on the RA test (20) where LoA ranged from 25% to −19% emphasizing the higher homogeneity of the sample in this study. When compared with other field tests, such as the YYIRT, the VO_2max_ estimation of the RA test was more precise (+4%). Previous research indicates that the YYIRT underestimates the VO_2max_ by 14% (34) to 30% (35). Considering the relation between estimated and measured VO_2max_, the RA test (r = 0.82) provides similar values when compared to previous research on the Cooper test (r = 0.87) (43), the 30-15 IFT (r = 0.69) (36) as well as the YYIRT which typically ranges at about r = 0.7 (37). Overall, the RA test calculation of VO_2max_ from total running time provides a useful estimation of aerobic performance that is in accordance with established field-based tests but should however still be interpreted as an indication rather than a prediction.

While the RA test appears to be suitable for the measurement of aerobic capacity, the comparisons with the RAST do not support its use in deriving anaerobic variables (FI, AC, PPOmax, PPOav). No correlation was observed for any of the variables most likely reflecting differences in metabolic demands between the RAST and RA test. The RAST consists of six short bouts, lasting approximately 8 s with intermittent 10 s breaks, which mainly addresses the phosphocreatine system (38). In contrast, the RA test comprises four 150 m sprints with multiple change of directions, averaging approximately 100 s per bout. According to the model by Gastin (39), a short exhaustive exercise with a duration of about 10 s is characterized by 96% of anaerobic energy supply while the aerobic system only contributes by about 4%. At 90 s exercise duration, this has shifted to 44% anaerobic and 56% aerobic. The metabolic crossover point where aerobic contribution begins to exceed anaerobic, occurs approximately around 75 s. Given the long duration per bout in the RA test, participants rely more on the oxidative metabolism. Therefore, although the RA test includes short sprints and change of direction which are associated with anaerobic energy metabolism (40), the long running distance reduces anaerobic contribution making it unsuitable to test anaerobic metrics captured by the RAST.

### Practical application

The study results support the use of multidirectional reactive agility protocols to determine key performance indicators for aerobic capacity in team ball sports. The developed RA test on the SKILLCOURT provides an alternative to existing assessments for directly measuring VO_2max_ and allows aerobic performance assessment in more ecologically valid environments. Importantly, after validating the RA test in a previous study (20), the results support the test's feasibility also for female-only athlete samples. Moreover, predicting aerobic capacity based on running time during the RA test was comparable to established tests such as the Cooper or 30-15 IFT. The combined pattern of result suggest that the RA agility test provides a promising tool for regular aerobic performance testing and potential use for talent identification in team ball sport athletes. However, while validity could be confirmed for aerobic capacity, this was not observed for anaerobic performance metrics. Therefore, the developed test in its current form should be used for the purpose of aerobic performance assessment only.

## Limitations and future directions

While the study supports the validity of the RA test on the SKILLCOURT to determine aerobic performance also in a homogeneous female athlete sample, future research needs to address the RA test's reliability to support its use for regular performance diagnostics. Further, additional analyses on the practical relevance of VO_2max_ estimation in the RA test are required. In an exploratory analysis, the results of the previous Cooper test and 30-15 IFT reflecting current team practice were included. However, these tests have been performed during different periods of the season. Although no substantial interactions between changes in aerobic capacity and player position are expected, these cannot be ruled out which might have been affected the comparison of correlation coefficients between RA test, Cooper test and 30-15 IFT. Future research needs to align the time points of measurement. While the study aimed to mitigate learning effects by familiarizing participants with the RA runs prior to the RA test, learning effects cannot be completely ruled out. However, this would have only affected the estimated but not the measured VO_2max_. Future research, should also determine athlete motivation as a potential influencing factor for VO_2max_ (41). Especially in female athlete this also applies to the menstrual cycle that may affect aerobic performance (42). Finally, research is warranted determining how well the performance in the RA test corresponds to performance indicators on the court (e.g., running distance, number of sprints, etc.) to substantiate its ecological validity.

## Conclusion

This study evaluated the validity of determining aerobic and anaerobic capacity using a novel reactive agility test in female handball players. The results indicate no differences in measured VO_2max_ between the RA test and a gold-standard treadmill protocol with reasonable limits of agreement. VO_2max_ estimation based on running time provided a good estimation comparable with existing Cooper and 30-15 tests but should still be considered an indication rather than prediction of aerobic capacity. In contrast, the test did does not allow determining anaerobic capacity. The RA test provides an ecologically valid alternative to existing laboratory and field-based tests to measure or estimate VO_2max_ and may be of particular interest for team ball sports.

## Data Availability

The raw data supporting the conclusions of this article will be made available by the authors, without undue reservation.
